# Diffuse preretinal infiltrates in a patient with orbital atypical T-cell lymphoproliferative infiltration masquerading posterior uveitis

**DOI:** 10.3205/oc000242

**Published:** 2024-09-23

**Authors:** Bilge Batu Oto, Oğuzhan Kılıçarslan, Didar Uçar, Samira Hagverdiyeva, Ahmet Murat Sarıcı

**Affiliations:** 1 Department of Ophthalmology, Cerrahpasa Medical Faculty, Istanbul University-Cerrahpasa, Istanbul, Turkey; 2 Department of Ophthalmology, Ayancık State Hospital, Sinop, Turkey; 3 Department of Ophthalmology, Faculty of Medicine, TC İstanbul Yeni Yüzyıl University, Istanbul, Turkey

**Keywords:** intraocular lymphoma, masquerade syndrome, orbital lymphoma, preretinal infiltrates, T-lymphocyte

## Abstract

**Purpose:**

To report an aggressive and rapidly progressive case of atypical T-cell lymphoproliferative infiltration both with intraocular and orbital involvement and preretinal infiltrates.

**Methods:**

Medical records and imaging of the patient were retrospectively reviewed.

**Case presentation:**

A 25-year-old woman presented first with preretinal infiltrates resembling uveitis and developed orbital and intracranial signs eventually during her evaluation. Clinical presentation worsened gradually. The patient developed bilateral proptosis, pupillary dilation and uvula deviation. Diagnostic orbital incision biopsy revealed T-cell lymphoproliferative disease.

**Conclusion:**

This case gives evidence that intraocular involvement due to T-cell lymphoproliferative disease may present as a masquerade syndrome and should be kept in mind in patients with extraordinary presentation.

## Background

Lymphoma constitutes an important part of ocular neoplasia. Intraocular and adnexal ocular lymphoma are presumed to be different entities in ophthalmology. For being one of the greatest masquerade syndromes in ophthalmology, intraocular lymphoma is a challenging diagnosis [1]. Sometimes it is quite hard to differentiate this entity from infectious or non-infectious posterior uveitis syndromes. Retinal, choroidal, and vitreous findings can present with similar features to infectious uveitis like syphilis and tuberculosis [2].

Adnexal lymphoma can reveal itself in periocular tissues such as conjunctiva, retro-orbital tissues, lacrimal gland, caruncula or eyelid. Each of these locations for neoplastic infiltration can develop different symptoms and clinical findings but not typical for a special type of ocular adnexal lymphoma. Histopathologic confirmation is essential both for diagnostic approach and proper evaluation of prognostic results. Specific molecular classification tests may play an important role to design the ideal therapy [3]. 

Herein, we report a case of atypical T-cell lymphoproliferative infiltration in orbita and vitreous which presented first like a masquerade syndrome with unique fundus and spectral-domain optic coherence tomography (SD-OCT) findings.

## Case description

A 25-year-old, Caucasian woman presented with headache and conjunctival hyperemia in the left eye twice in a 3 months period and each time she received topical treatment for conjunctivitis. Later, the patient developed visual loss and was referred to our clinic with the pre-diagnosis of uveitis.

**Figure 1:**
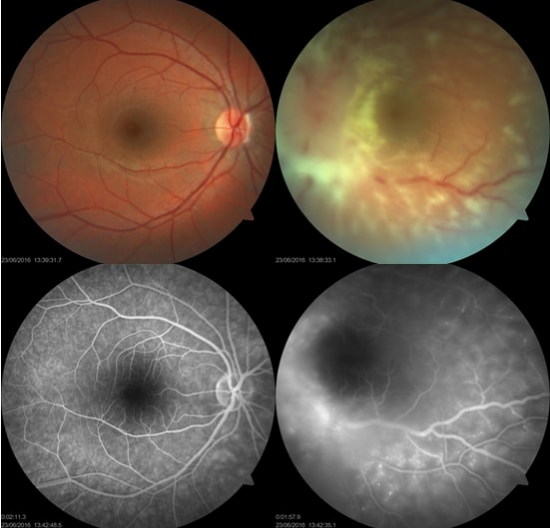
Fundus photographs in presentation are shown in upper images. Optic disc edema, vitreous haze +1, diffuse cotton wool spots were seen in the left eye. In fundus fluorescein angiography (lower images) optic nerve staining and vascular leakage around macula and arcades were observed in left eye.

**Figure 2:**
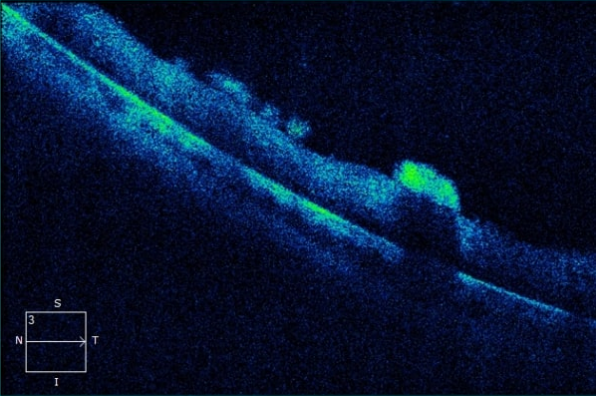
Peripheral preretinal infiltrates with different sizes in SD-OCT

At presentation, the patient had 20/20 visual acuity in the right eye and hand motion in the left eye. Direct light reflex was positive for both eyes and extraocular muscle movements were normal. In slit lamp examination, the right eye was normal and there were trace anterior chamber cells in the left eye. Right eye was normal in binocular indirect fundus examination and left eye revealed +1 vitreous haze along with severe papilledema and approximately 500 micron-width diffuse cotton wool spots on the retinal surface (Figure 1 (Fig. 1)). There were optic nerve staining and vascular leakage in fundus fluorescein angiography (FFA) in left eye (Figure 1 (Fig. 1)). Optical coherence tomography (OCT) of the left eye showed diffuse preretinal infiltrates (Figure 2 (Fig. 2)). 

Serologic tests including HbsAg, Anti-HIV, Anti-HCV, VDRL-RPR, and quantiferon were negative. The patient had a round density in the hilar region in chest X-ray resembling lymphadenopathy. Electrocardiography and cranial-orbital magnetic resonance imaging (MRI) were normal at first presentation. 

The patient was admitted to neurology service with suspicion of neurobrucellosis, tuberculosis meningitis or sarcoidosis. The serologic tests were found to be negative for Brucella IgM, Toxoplasma IgM, Herpes simplex type 1–2 IgM and CMV IgM. So, neurobrucellosis was ruled out and the patient was referred to rheumatologist for investigation of any vasculitis or autoimmune diseases. Rheumatologic test results were normal.

The patient had papilledema and clinical findings of increased intracranial pressure (headache, vomiting, neck stiffness), so lumbar puncture was performed after cranial tomography. Examination of cerebrospinal fluid (CSF) revealed low glucose and high protein levels. According to these CSF findings compatible with tuberculosis, treatment regimen for tuberculosis meningitis including isoniazid, rifampicin, ethambutol and pyrazinamide was commenced for the patient. Under this treatment regimen left lower hemifacial paralysis developed and the clinical signs of elevated intracranial pressure worsened. Because of the progression contrasted cranial MRI was performed and resulted normal. Pulse steroid treatment with 1 gr methylprednisolone was conducted to treat a possible neurosarcoidosis. Despite improving of headache and vomiting in first days, minimal response was observed with pulse steroid therapy. Angiotensin-converting enzyme (ACE) test and high-resolution computed tomography (HRCT) were in normal borders. CSF tuberculosis polymerase chain reaction (PCR) was also negative. Clinical course worsened under treatment and the patient developed bilateral proptosis, pupillary dilation and uvula deviation. 

**Figure 3:**
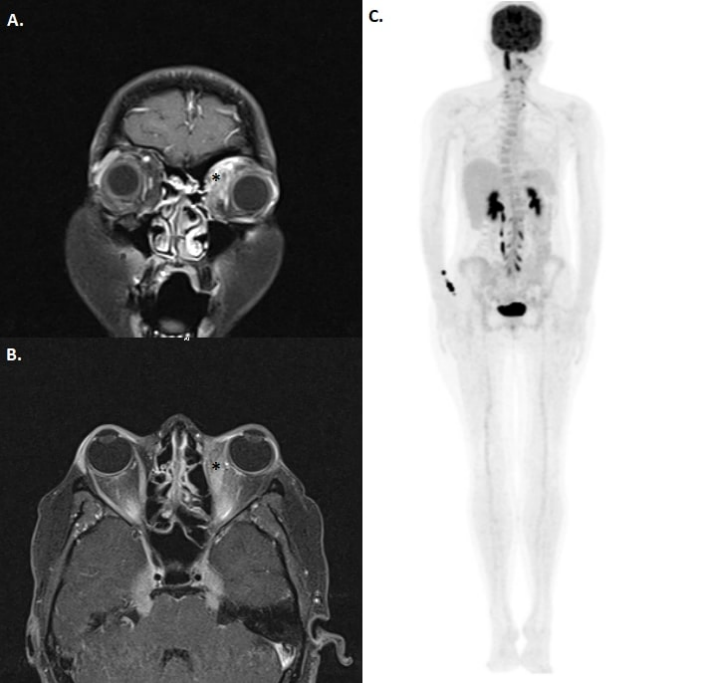
In coronal and axial T1 fat suppression MRI sections, increased contrast retention in left medial rectus and medial orbital soft tissue were observed (right). Increased FDG intake in right wrist, dorsal, lumbar and sacral vertebras was observed at PET-CT (left).

Repeated orbital magnetic resonance scanning with T1 fat suppression sections showed increased contrast retention in left medial orbital soft tissue (Figure 3A (Fig. 3)) and left medial rectus muscle (Figure 3B (Fig. 3)). Positron emission tomography (PET) scan was performed and increased fluorodeoxyglucose (FDG) uptake was seen in hypopituitary gland, left orbital medial region, right wrist, nasal septum, upper dorsal and lumbar vertebral and sacral region (Figure 3C (Fig. 3)). Anti-tuberculosis treatment was stopped due to disease progression under treatment and orbital biopsy was planned. 

An orbital biopsy from the medial region of the left eye was performed under general anesthesia. The disease had a rapid progression until the pathological results came out, paraparesis developed. Palliative radiotherapy was commenced before histopathologic diagnosis resulted because of suspicion of possible lymphocytic malignancy. The pathological examination of the orbital specimen revealed CD3 positive, atypical T-cell lymphoproliferative infiltration. 

While planning chemotherapy, the patient was taken to the intensive care unit after a cardiac arrest and died due to cardiopulmonary insufficiency 3 months after her initial presentation. 

## Discussion

Natural killer/T-cell lymphomas compromise less than 1.5% of all non-Hodgkin lymphomas, commonly seen in South East Asia. They are typically found in the nasal cavity and more than 95% are associated with the evidence of EBV infection [4]. Intraocular or adnexal lymphomas of non-B-cell type are rare and represent approximately 1% to 3% of all lymphoproliferative lesions in these sites [5], [6]. 

Intraocular lymphoma (IOL) is a rare condition and may be primary or due to systemic lymphoma with secondary ocular involvement. Primary intraocular lymphomas (PIOL) are the most common lymphoma of the eye and most of them are extranodal, non-Hodgkin, B-cell lymphomas. Intraocular T-cell lymphomas are uncommon; most of them represent secondary to metastasis of systemic T-cell lymphomas, including primary cutaneous peripheral T-cell lymphoma or other systemic T-cell lymphoma and have been regarded as markers of poor prognosis [4], [7].

PIOL is a major masquerade syndrome mimicking uveitis in ophthalmology practice. Presentation with anterior uveitis, retinal vasculitis, retinitis, and choroiditis has been reported [8], [9], [10], [11]. An orbital component can accompany PIOL or occur simultaneously. Cases with both ocular and orbital onset were reported in literature [12], [13]. Retinal findings due to systemic non-Hodgkin lymphoma can also be encountered [14]. 

Intraocular T-cell lymphomas are secondary to systemic lymphomas in most cases [15], [16], but there are several cases that have been reported with insidious ocular symptoms prior to systemic clinical presentation [17]. The most common ocular manifestation of intraocular T-cell lymphoma is vitritis followed by anterior uveitis [18]. Intraocular and orbital lymphoma presentation at the same time is quite a rare condition in ocular oncology. 

The present case had the typical clinical features of natural killer T cell lymphoma (NKTL) but described as both adnexal and intraocular involvement of atypical T-cell lymphoproliferative infiltration with specific preretinal infiltrates. Unfortunately, no histopathological examination for detailed cytological markers of T cell lymphoma was made. Okada et al. [19] reported a NKTL case both with adnexal and intraocular involvement in which the initial presentation was unilateral anterior uveitis refractory to topical treatment. In this case, MRI revealed a globe-surrounding mass in a non-tumoral appearance which misdirected the clinicians to orbital inflammatory disorder. After clinical deterioration with systemic pulse steroid therapy, orbital biopsy revealed the definitive diagnosis as a malignant lymphoma in this case. Our patient was a rare example of intraocular and ocular adnexal involvement of atypical T-cell lymphoproliferative infiltration, but fundus examination had more prominent findings. Papilledema, vitreous haze and diffuse preretinal infiltrates misled to an infectious posterior uveitic etiology. The presence of multiple cranial nerve palsies, especially neurobrucellosis, neurosarcoidosis, and tuberculosis, was investigated in our case initially. Due to the worsening of clinical symptoms under steroid treatment and development of bilateral proptosis, an infiltrative pathology was suspected and pathologic confirmation was obtained with incisional biopsy. 

The most specific feature in our case is the distinct appearance of diffuse preretinal infiltrates in fundus examination and SD-OCT. Besides, FFA findings were quite specific with diffuse perivascular hyperfluorescence. There are some reports about SD-OCT findings in intraocular lymphoma. Keino et al. [20] reported hyperreflective nodules in retina pigment epithelium, hyperreflective bands above retinal pigment epithelium (RPE), RPE elevations and disruption of ellipsoid zone as SD-OCT findings of IOL. 

## Conclusion

Both intraocular and adnexal involvement and the appearance of preretinal infiltrates make this case unique. Specific findings at SD-OCT and fundoscopy may be a significant diagnostic clue for intraocular T-cell lymphoid neoplasia.

## Notes

### Patient consent

Written informed consent was obtained from the patient for the publication of this case report and accompanying images.

### Competing interests

The authors declare that they have no competing interests.
